# The Function and Three-Dimensional Structure of a Thromboxane A_2_/Cysteinyl Leukotriene-Binding Protein from the Saliva of a Mosquito Vector of the Malaria Parasite

**DOI:** 10.1371/journal.pbio.1000547

**Published:** 2010-11-30

**Authors:** Patricia H. Alvarenga, Ivo M. B. Francischetti, Eric Calvo, Anderson Sá-Nunes, José M. C. Ribeiro, John F. Andersen

**Affiliations:** 1 Laboratory of Malaria and Vector Research, National Institutes of Health, National Institute of Allergy and Infectious Diseases, Rockville, Maryland, United States of America; 2 Laboratório de Bioquímica e Fisiologia de Artrópodes, Departamento de Química, Universidade Federal Rural do Rio de Janeiro, Seropédica, Brazil; 3 Laboratório de Imunologia Experimental, Departamento de Imunologia, Instituto de Ciências Biomédicas, Universidade de São Paulo, São Paulo, Brazil; Stanford University, United States of America

## Abstract

The highly expressed D7 protein family of mosquito saliva has previously been shown to act as an anti-inflammatory mediator by binding host biogenic amines and cysteinyl leukotrienes (CysLTs). In this study we demonstrate that AnSt-D7L1, a two-domain member of this group from *Anopheles stephensi*, retains the CysLT binding function seen in the homolog AeD7 from *Aedes aegypti* but has lost the ability to bind biogenic amines. Unlike any previously characterized members of the D7 family, AnSt-D7L1 has acquired the important function of binding thromboxane A_2_ (TXA_2_) and its analogs with high affinity. When administered to tissue preparations, AnSt-D7L1 abrogated Leukotriene C_4_ (LTC_4_)-induced contraction of guinea pig ileum and contraction of rat aorta by the TXA_2_ analog U46619. The protein also inhibited platelet aggregation induced by both collagen and U46619 when administered to stirred platelets. The crystal structure of AnSt-D7L1 contains two OBP-like domains and has a structure similar to AeD7. In AnSt-D7L1, the binding pocket of the C-terminal domain has been rearranged relative to AeD7, making the protein unable to bind biogenic amines. Structures of the ligand complexes show that CysLTs and TXA_2_ analogs both bind in the same hydrophobic pocket of the N-terminal domain. The TXA_2_ analog U46619 is stabilized by hydrogen bonding interactions of the ω-5 hydroxyl group with the phenolic hydroxyl group of Tyr 52. LTC_4_ and occupies a very similar position to LTE_4_ in the previously determined structure of its complex with AeD7. As yet, it is not known what, if any, new function has been acquired by the rearranged C-terminal domain. This article presents, to our knowledge, the first structural characterization of a protein from mosquito saliva that inhibits collagen mediated platelet activation.

## Abstract 2

A salivary protein from a malaria-transmitting mosquito uses a single domain to bind to thromboxane A_2_ and cysteinyl leukotrienes and prevent blood clotting and inflammation in the host on which it feeds.

## Author Summary

When feeding, a female mosquito must inhibit the blood clotting and inflammatory responses of the host. To do this, the insect produces salivary proteins that neutralize key host molecules participating in clotting and inflammation. Here, we describe a salivary protein AnSt-D7L1 that scavenges both thomboxane A_2_ and cysteinyl leukotrienes, two substances involved in blood vessel constriction, platelet aggregation, and inflammatory responses to an insect bite. We produced this protein in bacteria and showed that it tightly binds both these molecules, inhibiting the processes in which they are involved. We then determined its structure using X-ray crystallography and showed that there is a single binding site in one domain of the protein, accommodating both thromboxane A_2_ and cysteinyl leukotrienes, and that this site is responsible for the scavenging effect of the protein. These studies reveal the structural features of proteins needed to bind to key molecules of potential pharmacological importance and add to our understanding of the process of mosquito blood feeding, which is essential for transmission of the malaria parasite.

## Introduction

Hematophagous arthropods produce a varied mix of salivary proteins, peptides, and small molecules aimed at overcoming the hemostatic and inflammatory responses of the host. In order to successfully take a meal, the blood feeder must prevent host vasoconstrictive responses and the clotting of blood as it travels through the mouthparts to the gut [1],[2]. The inhibition of immediate inflammatory responses is also essential, in that the swelling, itching, and pain resulting from arthropod bites may themselves interfere with the ingestion of blood or elicit defensive behavioral responses from the host [1]–[4]. Additionally, inflammation in the skin at the site of feeding has been shown to influence the establishment of infection by arthropod-vectored pathogens, making the anti-inflammatory components of saliva important from this standpoint as well [5],[6]. In fact, several pathogens take advantage of the biological properties of the salivary mixture to infect their hosts and cause disease. It has been demonstrated that immunity against salivary components from different mosquito species is able to reduce disease transmission by these vectors [7]–[10]. In addition, the mosquito life cycle is affected by immunization against salivary molecules [11].

Wounding as a result of a mosquito bite exposes collagen and other matrix proteins that act to initiate the activation of platelets. The stimulation of TXA_2_ biosynthesis and the release of dense granules containing small molecule mediators of platelet activation and inflammation such as ADP and serotonin potentiate the activation response. In sensitized hosts, IgE antibodies recognizing salivary proteins activate mast cells in the skin, leading to the release of large amounts of histamine and the synthesis of CysLTs [1],[2]. These compounds cause rapid swelling, pain, increases in vascular permeability, and itching in the host. Numerous proteins have now been identified in the salivas of blood feeders that act to limit the responses of platelets and mast cells to arthropod bites [12]–[19].

An important functional theme in the physiology of blood feeding is the use of specific salivary binding proteins to sequester small-molecule agonists of inflammation and platelet activation [14],[17],[18],[20]. Since hematophagy has evolved independently many times in insects and other arthropods, proteins from different structural families act to perform these functions in the various blood-feeding species [1],[2],[13]. To generate the molecular diversity needed to bind small molecule effectors of widely varying structure, multigene families have arisen from gene duplication events, with the resulting gene products being expressed specifically in the salivary glands of blood feeders [21]. Individual members of these families have diverged in sequence and structure, resulting in an array of protein forms with different ligand binding specificities. In some instances, salivary proteins have acquired the ability to form specific interactions with host proteins while maintaining the ability to bind small molecule effectors. The diversity of these binding protein families may reflect an evolutionary arms race between the arthropod blood feeders and the sophisticated hemostatic, inflammatory, and immune systems of their vertebrate hosts.

Two protein families have thus far been implicated as scavengers of small molecule modulators of inflammation and platelet aggregation in blood feeding arthropods. The lipocalins serve this function in ticks and triatomine bugs, while members of the D7 family serve the function in mosquitoes [14],[20],[22]. D7 proteins are structurally related to the arthropod OBPs and come in two forms. The long form proteins, as exemplified by AeD7 from the saliva of *Aedes aegypti*, have two OBP domains, with each domain binding a single ligand molecule [14]–[16]. Single-domain D7 proteins are found in mosquitoes and other members of the Nematocera suborder of flies, with those from *An. gambiae* (referred to as D7r proteins) being extensively characterized and found to bind a single ligand molecule per protein [14]. A group of proteins orthologous to D7r is found in *An. stephensi*, and one of these has also been found to function as an inhibitor of coagulation and inflammation pathways by binding factor XII(a) and high molecular weight kininogen [23]. The functionally equivalent ortholog of this molecule has been identified in *An. gambiae* as the protein D7r1 [14]. Both AeD7 and the D7r proteins bind the biogenic amines serotonin, norepinephrine, and histamine with high affinity. The C-terminal domain of AeD7 is responsible for biogenic amine binding and is homologous to the single domain D7r proteins, while the N-terminal domain is distinct in sequence and structure and is a scavenger of CysLTs [15]. Despite the presence of the highly expressed, biogenic amine-binding, short form D7 proteins in all species of *Anopheles* examined, the two-domain long forms of D7 have not been lost, suggesting that they may play, at least in part, a different role in these species.

In this study we examine the ligand binding specificity, physiological function, and three dimensional structure of AnSt-D7L1, a two-domain D7 protein from *An. stephensi*, a vector of the malaria parasite. We found that unlike other members of the D7 family, this protein functions as a TXA_2_-binding protein, thereby acting to inhibit the activation of platelets during feeding. We also describe the crystal structures of ligand complexes that provide, to our knowledge, the first view of the molecular interactions involved in TXA_2_ binding. We ask whether evolution of the short D7 proteins in *Anopheles sp*. and their assumption of the biogenic amine binding function otherwise seen in the long form of *Aedes* has resulted in the evolution of functional changes in the protein. We find that AnSt-D7L1 has acquired the ability to bind the vasoactive platelet aggregation and proinflammatory agonist TXA_2_ in addition to maintaining the ability to bind CysLTs. Additionally, the C-terminal domain has undergone extensive structural rearrangements resulting in a loss of the biogenic amine binding function.

## Results

### AnSt-D7L1 a Two-Domain D7 Protein

**Figure 1:**
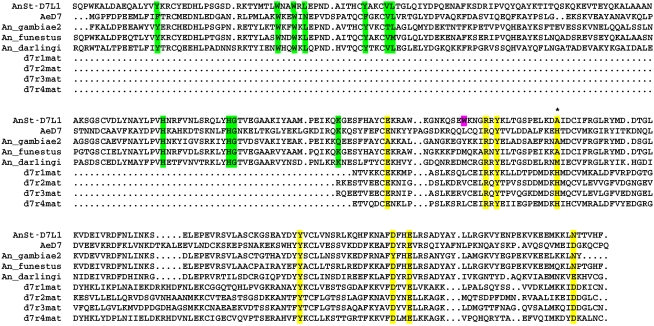
Amino acid sequence alignment of D7 proteins from *Anopheles* sp. and *Ae. aegypti*. The alignment shows sequences of the two-domain D7 forms from *An. stephensi* (AnSt-D7L1), *An. gambiae*, *An. funestus*, and *An. darlingi* in comparison to the two-domain protein from *Aedes aegypti* (AeD7). Also shown are the four single-domain D7 proteins (D7r1–4) from *An. gambiae*. Conserved residues contained in the ligand binding pocket of the N-terminal domain are highlighted in green, while those in the biogenic amine binding pocket of AeD7 and *Anopheles* single-domain D7s are highlighted in yellow. In the single-domain proteins and in the C-terminal domain of the two-domain proteins the conserved histidine residue (His 189 of AeD7) involved in biogenic amine binding is marked with an asterisk. Also highlighted in magenta is Trp 173 of AnSt-D7L1. In biogenic amine-binding forms, this residue is either leucine or valine.

The amino acid sequence of AnSt-D7L1 is 31% identical to that of AeD7, the two-domain D7 from *Ae. aegypti* (Figure 1). AeD7 contains binding sites for both CysLTs and biogenic amines, with the CysLT binding site lying in the N-terminal domain and the biogenic amine binding site in the C-terminal domain. Residues lining the CysLT binding pocket in the N-terminal domain of AeD7 are conserved to a large degree in AnSt-D7L1 and other two-domain D7 proteins from *Anopheles* sp. (Figure 1). The C-terminal domain of AnSt-D7L1 also shows conservation of most but not all of the residues from the biogenic amine binding pocket in AeD7 and the D7r proteins from *Anopheles* species. Notably, the position of His 189 in AeD7 contains alanine (Ala 190) in AnSt-D7L1 and other two-domain D7 proteins from *Anopheles* species (Figure 1). This residue is highly conserved in biogenic amine-binding D7 proteins where it forms a hydrogen bond with the phenolic hydroxyl groups of serotonin and catecholamines [15],[16].

### AnSt-D7L1 Binds CysLTs

In order to determine the role of AnSt-D7L1 in blood feeding, we examined its ligand binding properties. A series of ITC experiments were performed to screen potential candidate ligands, including bioactive lipids, biogenic amines, and nucleotides that are known to be involved in inflammation and hemostasis (Figure S1).

**Figure 2:**
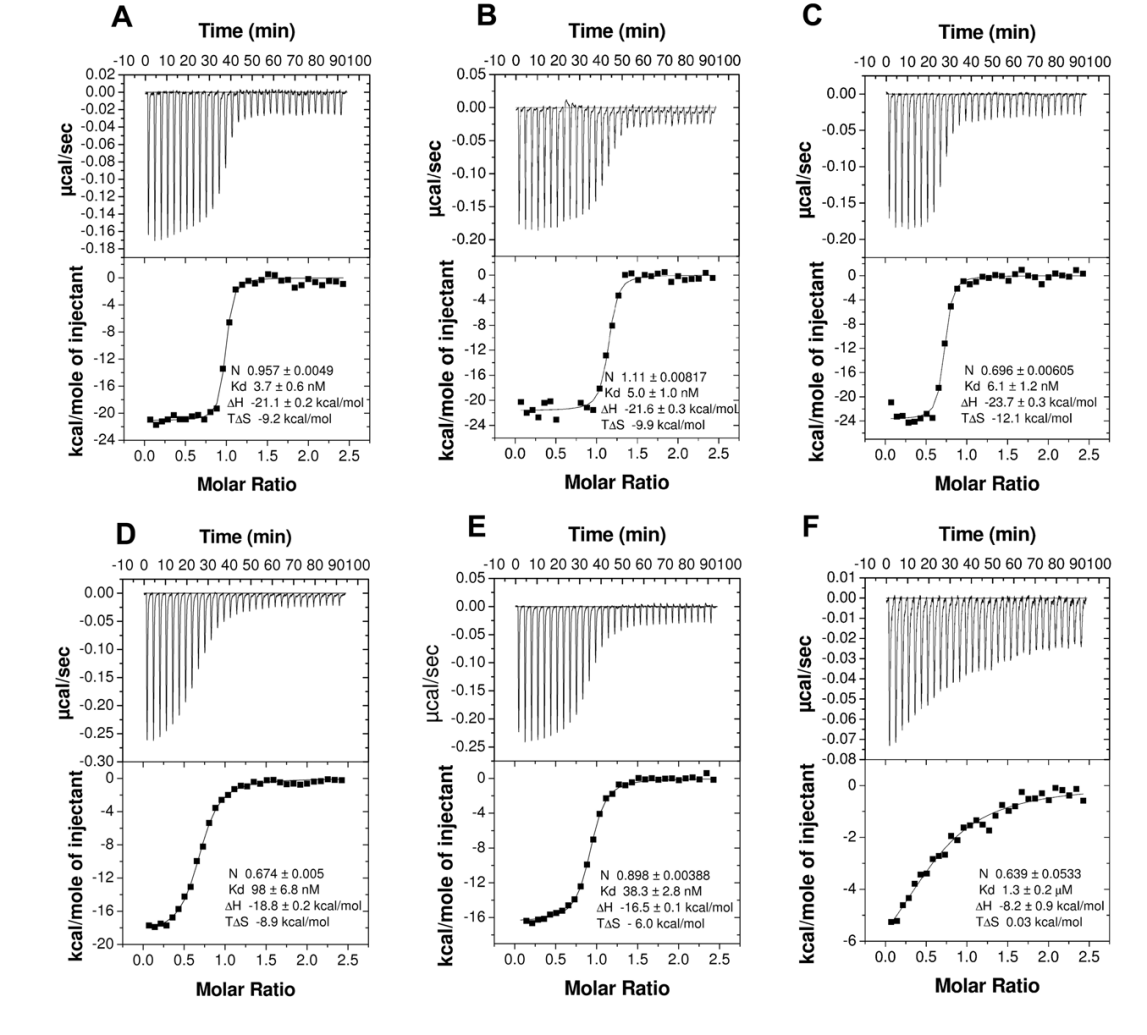
Binding of CysLTs and TXA_2_ analogs by ITC. Binding experiments were performed on a MicroCal VP-ITC instrument. For CysLT experiments the syringe contained a 20 µM ligand solution, and the AnSt-D7L1 concentration in the cell was 2 µM. For the TXA_2_ analog experiments the ligand solution used to fill the syringe was 40 µM and the AnSt-D7L1 concentration in the cell was 4 µM. Assays were performed at 35°C with successive 10 µL injections. The upper curve in each panel shows the measured heats for each injection, while the lower plot shows the enthalpies for each injection along with the fit to a single binding site model used to estimate the dissociation constant (Kd), enthalpy of binding (ΔH), entropy of binding (TΔS), and the binding stoichiometry (N). Panels: (A) LTC_4_, (B) LTD_4_, (C) LTE_4_, (D) U46619, (E) carbocyclic TXA_2_, (F) TXB_2_.

AnSt-D7L1 bound the CysLTs LTC_4_, LTD_4_, and LTE_4_ with a stoichiometry of one ligand molecule per molecule of protein (Figure 2A–C). The binding affinities for CysLTs were approximately 10 times higher than those previously reported for the related protein AeD7 [15]. As in AeD7, the affinities for the three CysLTs are similar to one another, suggesting that the protein recognizes the lipid portion of the ligand, rather than the peptide portion. AnSt-D7L1 did not bind LTB_4_, a ligand previously shown to bind with AeD7 [15] with low affinity (unpublished data).

### AnSt-D7 L1 Binds Thromboxane A2 Analogs

The eicosanoid TXA_2_ is an important mediator of platelet activation and vascular tone. Because of its instability, TXA_2_ binding could not be evaluated directly using ITC. In solution, it undergoes rapid hydrolysis to form TXB_2_, a stable but physiologically inactive compound. Binding with stable analogs could be measured, however, with U46619 and carbocyclic TXA_2_ exhibiting dissociation constants of approximately 98 nM and 38 nM, respectively (Figure 2D,E). This suggested that TXA_2_ itself may be a physiological target of AnSt-D7L1. The binding affinity for TXB_2_, the major hydrolysis product of TXA_2_, was approximately 13-fold lower than for U46619, demonstrating that the protein may be able to discriminate between the active and inactive thromboxane forms in vivo (Figure 2D,F).

### AnSt-D7L1 Does Not Bind Biogenic Amines, ADP, or PAF

AeD7 and the single-domain D7r proteins of *An. gambiae* have been characterized as scavengers of the biogenic amines serotonin, norepinephrine, and histamine [14]–[16]. AnSt-D7L1 showed no detectable binding of biogenic amines, however, suggesting that its C-terminal domain is structurally distinct from the comparable, biogenic amine-binding C-terminal domain of AeD7 (unpublished data). We have also tested the binding of AnSt-D7L1 to the platelet activating phospholipid derivative PAF and also to the nucleotide ADP. Neither showed any detectable binding (unpublished data).

### AnSt-D7L1 Shows Weak Binding with Prostaglandins and Does Not Bind Arachidonic Acid

**Figure 3:**
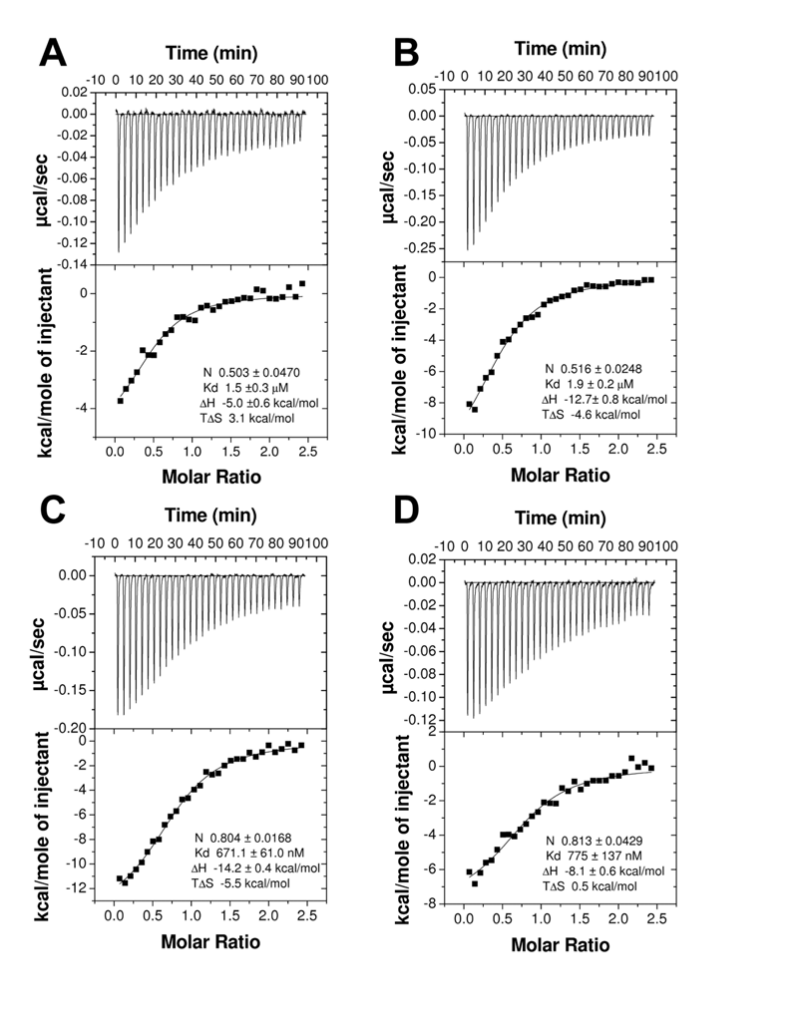
Binding of AnSt-D7L1 to prostaglandins and prostaglandins analogues by ITC. Binding experiments were performed on a MicroCal VP-ITC instrument. For these experiments, the ligand solution used to fill the syringe was 80 µM and AnSt-D7L1 concentration in the calorimeter cell was 8 µM. Assays were performed at 35°C with successive 10 µL injections. Upper curve in each panel shows the heat for each injection; lower part shows the enthalpies for each injection and data were fit to a single binding site model used to calculate the binding stoichiometry (N), dissociation constant (Kd), enthalpy of binding (ΔH), and entropy of binding (TΔS). Panels: (A) PGD_2_, (B) PGE_2_, (C) PGF_2α_, (D) U51605.

To determine if AnSt-D7L1 is specific in its binding for CysLTs and TXA_2_, we screened a number of additional eicosanoid compounds using ITC. AnSt-D7L1 showed no detectable binding to AA (unpublished data) but bound the prostaglandins PGD_2_, PGE_2_, and PGF_2α_ with affinities much lower than those seen with CysLTs and TXA_2_ analogs (Figure 3). The PGH_2_ analog U51605 also showed detectable but much weaker binding than U46619 (Figure 3D). This compound is also similar in structure to U46619 and carbocyclic TXA_2_ but lacks the hydroxyl group at the ω-5 position of the hydrocarbon chain, suggesting that this moiety plays an important role in the binding of TXA_2_ analogs.

### AnSt-D7L1 Binding to LTC_4_ Does Not Modify Bound CysLTs

To test for a hydrolytic or enzymatic function of AnSt-D7L1, LTC_4_ was incubated with the protein at 25°C and the mixture was analyzed by HPLC and mass spectrometry. Analytical gel filtration chromatography revealed only one peak corresponding in molecular size to the protein, indicating the formation of a leukotriene complex (Figure S2). Fractions containing the complex were then applied to a C18 HPLC column, eluted with methanol, and analyzed by mass spectrometry. The spectrum of the bound ligand was essentially identical to that of the free ligand, indicating that the protein binds but does not chemically modify LTC_4_ (Figure S3).

### AnSt-D7L1 Inhibits Agonist-Induced Smooth Muscle Contraction

**Figure 4:**
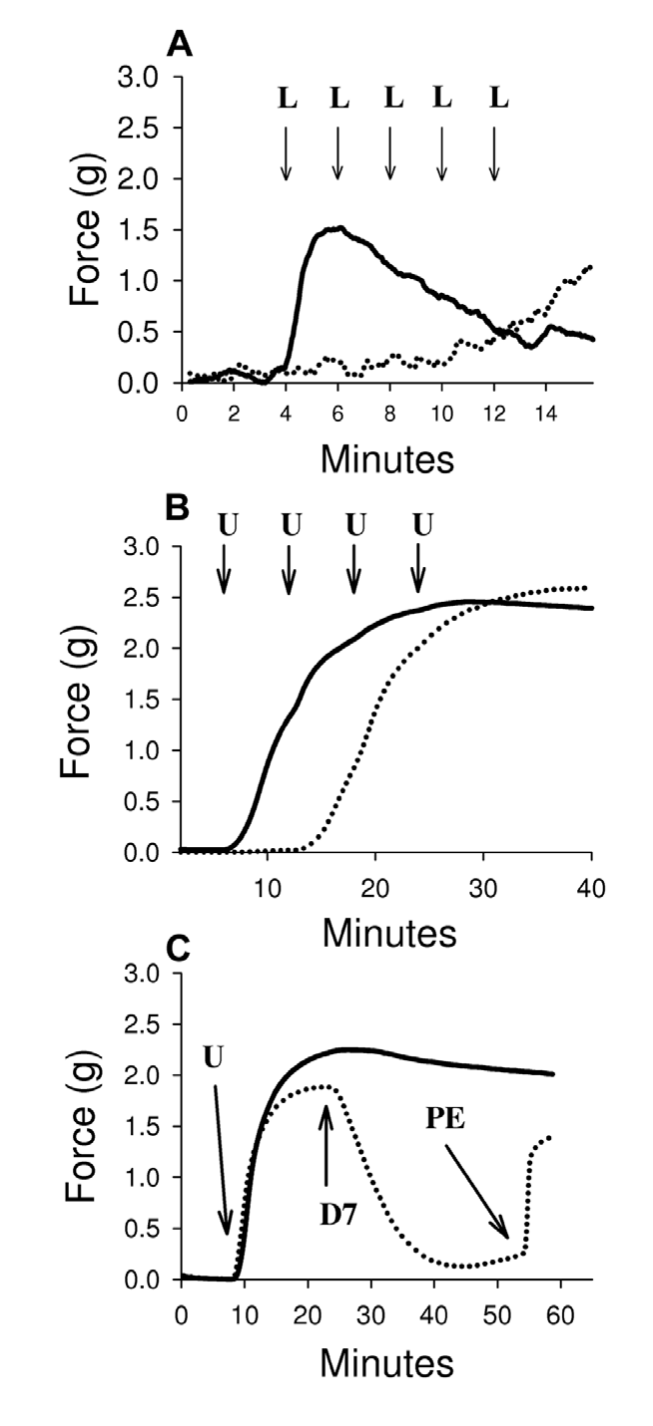
Smooth muscle contraction bioassay. (A) Guinea pig ileum samples were pre-incubated with buffer (solid line) or 1 µM AnSt-D7L1 (dotted line) and contraction was induced by 0.1 µM LTC_4_ (L), as indicated by the far left hand arrow, in both control and experimental preparations. Since in the presence of AnSt-D7L1 no contraction was observed after first LTC_4_ addition, further successive additions of 0.1 µM LTC_4_ were made only to this preparation (dotted line), as indicated by additional arrows. (B) Rat aorta samples were pre-incubated with buffer (solid line) or AnSt-D7L1 (dotted line) and the effect of addition of 0.1 µM U46619 (U) was recorded. Since there was no contraction in the presence of the AnSt-D7L1 after the first addition, further additions of 0.1 µM U46619 were made only to the preparation incubated with protein, as indicated by additional arrows. (C) The ability of AnSt-D7L1 to reverse contraction induced by U46619 was tested by pre-contacting rat aorta samples with 1 µM U46619 (as indicated by the letter “U”). After the plateau was achieved, in the experimental sample (dotted line) 0.1 µM of AnSt-D7L1 was added (indicated by D7 in the figure), which caused relaxation of the sample. In order to show that the muscle was still functional, 3 µM phenylephrine was added (indicated by PE). A control (solid line) experiment was performed without addition of protein or PE in order to show that in the absence of AnSt-D7L1 contraction lasts throughout the experiment.

One of the well-characterized physiological effects of CysLTs and TXA_2_ is the induction of smooth muscle contraction [24], including pulmonary vascular smooth muscle [25], guinea pig ileum [26], and aorta [27]. AnSt-D7L1 effectively abrogated LTC_4_-induced contraction (Figure 4A) of ileum preparations, demonstrating the ability of the protein to sequester ligand molecules and prevent them from interacting with their cognate receptors. When administered to a preparation of rabbit aorta, AnSt-D7L1 also inhibited U46619-induced contraction, increasing by approximately 3-fold the amount of U46619 needed to promote contraction in the absence of the protein (Figure 4B). AnSt-D7L1 was also able to reverse contraction induced by U46619 when added after the agonist (Figure 4C). In order to verify that the protein had no additional effect on the integrity of aorta preparations, phenylephrine (PE), an α1-adrenergic receptor agonist, was added to the preparation after relaxation by AnSt-D7L1. The aorta contracted normally, further verifying that the effect of AnSt-D7L1 was due only to its ability to bind U46619 (Figure 4C).

### AnSt-D7L1 Inhibits Collagen-Induced Platelet Aggregation

Exposure of collagen from the subendothelial matrix at the site of a wound plays a key role in triggering platelet adhesion and activation. Platelet interaction with collagen may be indirect, due to binding of the VWF-collagen complex with glycoprotein Ib-IX-V (GPIb-IX-V) or α_IIb_β_3_, or direct, via interaction with the platelet collagen receptors α_2_β_1_ (or glycoprotein Ia/IIa) and glypoprotein VI (GPVI) [28]–[32]. These events lead to the synthesis of TXA_2_ and release of ADP, which act together with collagen to amplify pro-aggregatory signals resulting in “inside out” activation of integrin α_IIb_β_3_, the receptor for fibrinogen and fibrin, and consequent platelet aggregation [29],[32]. At higher concentrations, collagen acts as a strong agonist of GPVI leading to platelet activation and aggregation in a manner that is independent of secretion of TXA_2_ or ADP [31],[32]. At low concentrations of collagen, however, aggregation depends on amplification of the response by TXA_2_ and ADP [31].This being the case, we hypothesized that binding of TXA_2_ by AnSt-D7L1 should inhibit platelet activation by low concentrations of collagen and have no effect at higher collagen concentrations.

**Figure 5:**
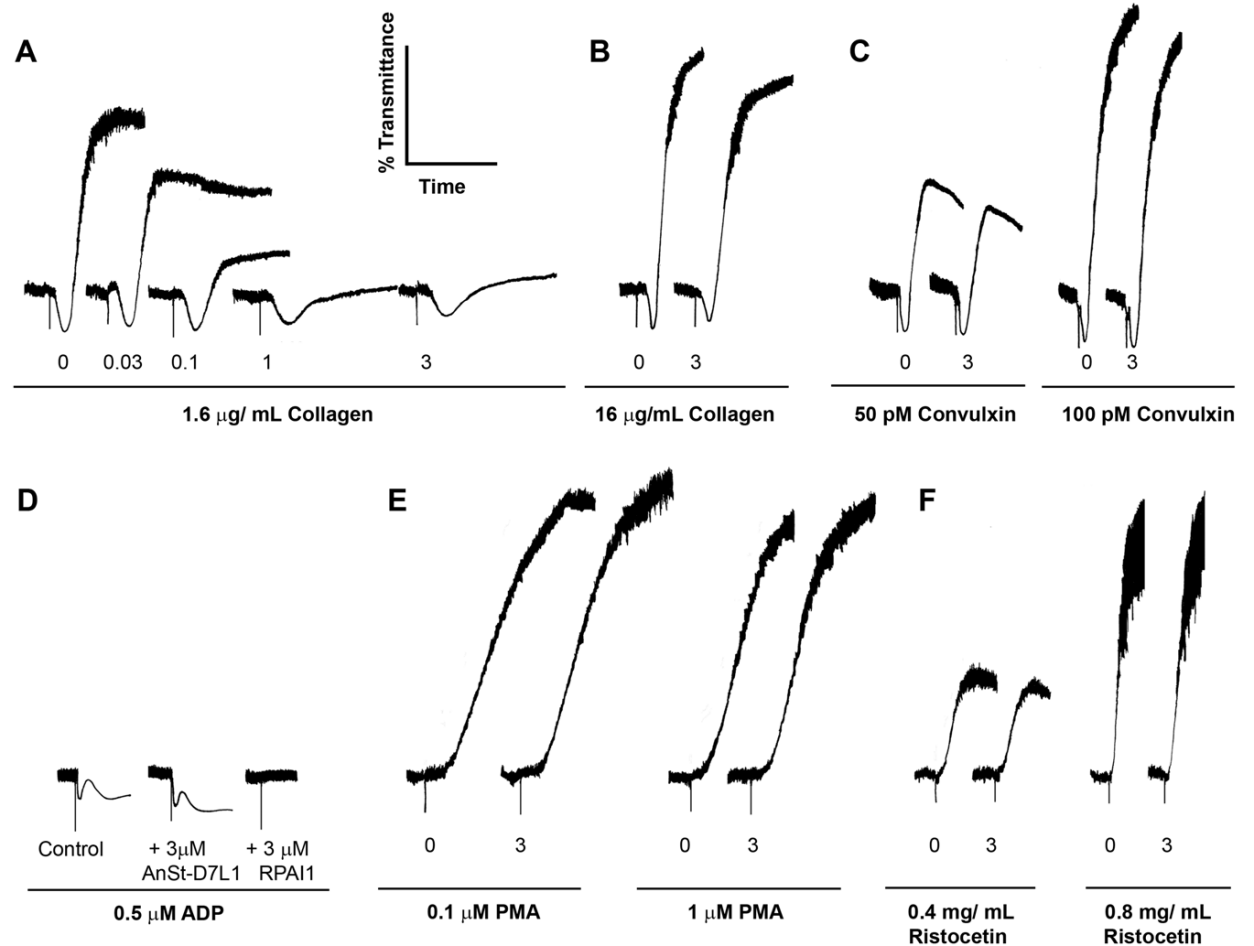
Effect of AnSt-D7L1 on platelet aggregation promoted by different agonists. Platelet-rich human plasma was incubated with different concentrations of AnSt-D7L1 for 1 min prior to the addition of agonists as indicated. Aggregation was measured at 37°C on an aggregometer. Unless otherwise stated protein concentration (µM) is shown under respective curves. (A) Concentration dependence of AnSt-D7L1 inhibition of collagen-mediated platelet aggregation at low concentration of collagen. (B) Lack of inhibition of collagen-mediated platelet aggregation by AnSt-D7L1 at high concentration of collagen. (C) Lack of inhibition of convulxin-mediated platelet aggregation by AnSt-D7L1. (D) ADP-mediated platelet aggregation in the absence of any protein (control), in the presence of 3 µM AnSt-D7L1, or in the presence of 3 µM RPAI1, which is known to bind ADP. (E) Lack of effect of AnSt-D7L1 on PMA-induced platelet aggregation. (F) Lack of effect of AnSt-D7L1 on ristocetin-induced platelet aggregation.

When we incubated stirred platelets with AnSt-D7L1 and stimulated with a low concentration (1.6 µg/mL) of collagen, we observed a delay in the onset of shape change and a diminished extent of platelet aggregation (Figure 5A). The degree of inhibition was dependent on the concentration of AnSt-D7L1, and under these conditions, aggregation was almost completely inhibited by a concentration of 1 µM AnSt-D7L1 (Figure 5A). Conversely, when platelets were stimulated by collagen at high concentration (Figure 5B) or by the potent GPVI agonist convulxin (Figure 5C), the inhibitory effect of AnSt-D7L1 was lost.

Platelet aggregation induced by collagen is also affected by apyrase, indicating an important role for ADP. Shape change induced by lower concentrations of ADP is a TXA_2_-independent process, however, and should not be limited by an inhibitor targeting TXA_2_ [33]. When we administered the minimal dose of ADP needed to elicit shape change to stirred platelets, preincubation with AnSt-D7L1 had no inhibitory effect (Figure 5D). This indicates that AnSt-D7L1 does not affect ADP signaling directly. Conversely, RPAI1, a specific ADP-binding protein from *Rhodnius prolixus* saliva [22],[34], was able to completely inhibit the shape change response under these conditions (Figure 5D). AnSt-D7L1 also showed no effect on platelet aggregation induced by the protein kinase C activator PMA (Figure 5E) or on ristocetin-induced platelet agglutination, which is dependent on VWF (Figure 5F). Both of these pathways are independent of TXA_2_, suggesting that the inhibition of collagen-mediated aggregation by AnSt-D7L1 depends only on its ability to bind TXA_2_.

### AnSt-D7L1 Mimics the Effects of TXA_2_ Receptor Antagonists or Inhibitors of Biosynthesis

**Figure 6:**
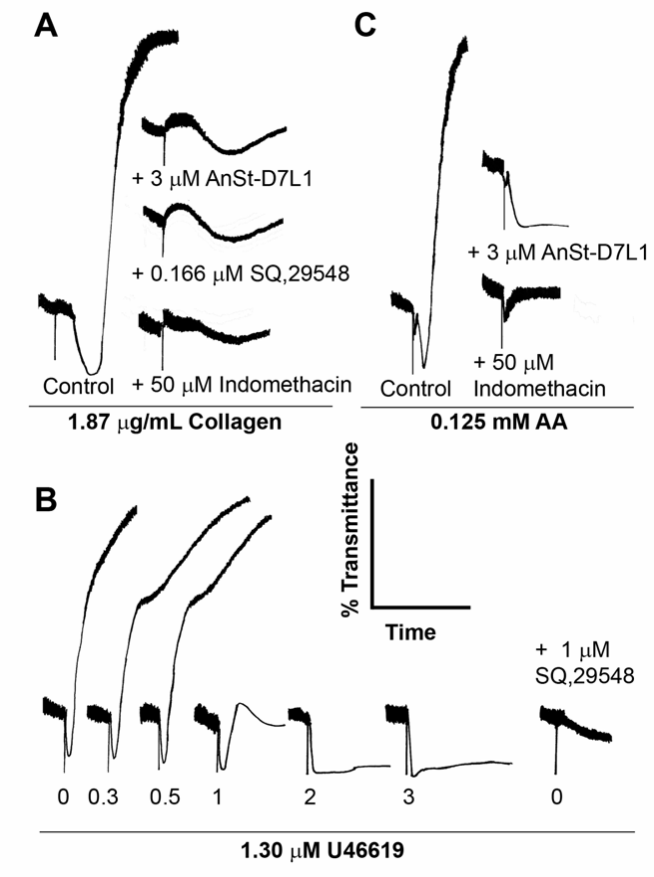
AnSt-D7L1 affects platelet aggregation promoted by TXA_2_. Platelet-rich human plasma was incubated with different concentrations of AnSt-D7L1 or buffer, as indicated, for 1 min prior to the addition of agonists indicated in each panel. (A) Aggregation mediated by collagen in the presence of TXA_2_ antagonists. The effect of 3 µM AnSt-D7L1 on collagen-induced aggregation was similar to the effect promoted by a thromboxane receptor (TP) antagonist (SQ 29,548) or by pre-incubating platelets with indomethacin, a general cyclooxygenase inhibitor. The curve marked control shows the normal response of platelets to collagen. (B) AnSt-D7L1 has a dose dependent effect on U46619-induced platelet aggregation. Protein concentrations are shown on the bottom of each curve. The last curve in the panel shows the effect of 1 µM SQ 29,548 in the absence of AnSt-D7L1. (C) AnSt-D7L1 (3 µM) affects AA-induced platelet aggregation. A similar effect is observed when platelets are pre-incubated with indomethacin in the absence of protein, inhibiting TXA_2_ production. Respective treatments are indicated in the bottom of each curve.

When platelets were activated by collagen in the presence of SQ 29,548, an antagonist of TXA_2_ receptor (TP), or were pre-treated with indomethacin, an inhibitor that prevents TXA_2_ biosynthesis, aggregation was attenuated (Figure 6A). The effect obtained with both treatments was very similar to that seen with 3 µM AnSt-D7L1 (Figure 6A), further supporting the idea that the activity of the protein is due to its ability to bind TXA_2_. This result is important in showing that AnSt-D7L1 is able to bind TXA_2_ itself, since in ITC experiments we were limited to the testing of stable analogues of TXA_2_.

### AnSt-D7L1 Inhibits Activation of Platelets by TXA_2_ Analogs and Biosynthetic Precursors

AnSt-D7L1 inhibited U46619 (1.3 µM)-induced platelet aggregation in a concentration-dependent manner (Figure 6B). At low concentrations of AnSt-D7L1 (less than a 1∶1 ratio of protein to inhibitor), the protein delayed but did not prevent platelet aggregation. As the ratio approached 1∶1 (at 1 µM AnSt-D7L1), the protein dramatically inhibited aggregation, an observation consistent with the 1∶1 binding stoichiometry seen in ITC experiments (Figure 2D).

Although higher concentrations (2 and 3 µM) of the protein completely prevented aggregation of platelets, shape change was not completely inhibited and could only be prevented by blockade of the TP receptor with the antagonist SQ 29,548 (Figure 6B). This is possibly due to the fact that TP-mediated shape change is coupled to a Gα_12/13_ protein signaling cascade, which requires only small amounts of U46619 (EC_50_ around 13 nM) [27],. Larger amounts of U46619 are necessary to promote aggregation, which is dependent on Gα_q/11_-coupled signaling [27],[35].

Since AA is a biosynthetic precursor of TXA_2_, as well as of prostaglandins, we tested the effect of AnSt-D7L1 on platelet aggregation induced by this compound. AnSt-D7L1 drastically decreased platelet aggregation induced by AA, showing a similar effect to that observed when platelets were pre-treated with indomethacin (Figure 6C). Since ITC data indicated that this protein does not bind AA itself (unpublished data), we conclude that the observed effect is due to AnSt-D7L1 binding of newly synthesized TXA_2_.

### Crystal Structures of AnSt-D7L1: Ligand-Free Protein

**Figure 7:**
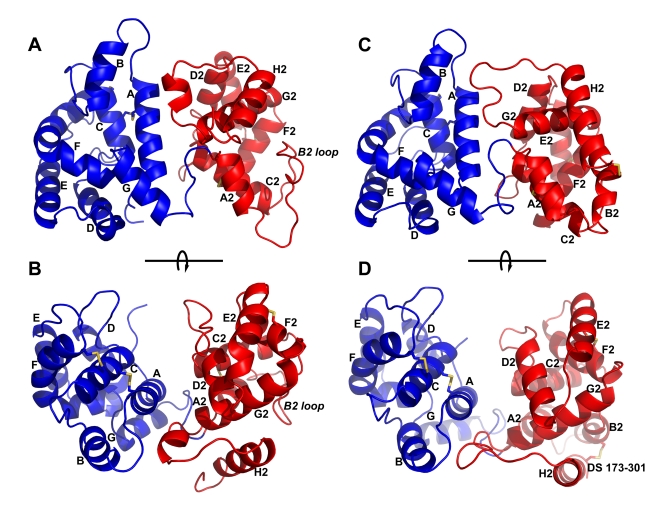
Ribbon diagram of the AnSt-D7L1 structure and comparison with AeD7. Structures of AnSt-D7L1 and AeD7 have the N-terminal domain colored blue and the C-terminal domain colored red. Cysteine residues forming disulfide bonds are shown in stick representation and colored yellow. (A) and (B) are two views of AnSt-D7L1 related by an approximately 90° rotation around the axis shown. (C) and (D) are two views of AeD7 from *Ae. aegypti* oriented in the same manner as (A) and (B). The helices are labeled A–G in the N-terminal domain and A2–H2 in the C-terminal domain. Helix B2 is missing in AnSt-D7L1, but the labeling of the other C-terminal domain helices corresponds to AeD7. The label “B2 loop” in (A) and (B) indicates the portion of the structure corresponding to α-helix B2 in AeD7. The label “DS 173–301” marks the position of the disulfide bond linking α-helix H2 with α-helix B2 in AeD7. The cysteine residues forming this bond are not present in AnSt-D7L1 (see panel B for comparison).

In order to characterize the ligand binding sites and understand the relationships and evolution of proteins in the D7 family, we determined the crystal structure of AnSt-D7L1 in the absence of ligands and in the presence of U46619 and LTC_4_ (Table 1). The ligand-free form of the protein crystallized in the space group P2_1_2_1_2_1_ with one protein molecule in the asymmetric unit. The structure revealed two OBP domains with the N-terminal domain containing three disulfide bonds and the C-terminal domain containing two (Figure 7A,B). The interface between the domains contains a number of residues that interact by hydrogen bonding or cation pi interactions. In its overall structure, the protein is similar to AeD7 with an RMS deviation of 1.82 Å over 239 Cα positions (Figures 1, 7).

**Table 1:** Data collection, phasing, and refinement statistics for AnSt-D7L1 and its ligand complexes.

Crystal	Selenomethionine	Unliganded	U46619	LTC_4_
Resolution (Å)	25.0–2.00	50.0–1.77	24.0–1.45	50.0–1.43
Beamline	22-BM	22-BM	19-BM	19-BM
Wavelength (Å)	0.97242	0.97242	0.97910	0.97910
Completeness	99.6/95.9	99.6/96.1	99.6/95.6	99.5/96.0
Average redundancy (total/high resolution shell)	8.8/5.9	10.0/10.1	4.7/4.3	4.7/3.5
R_merge_ (total/high resolution shell)	14.0/63.9	5.3/10.0	4.0/17.9	4.3/30.3
I/sigI (total/high resolution shell)	6.7/2.3	32.2/31.9	22.8/9.0	16.7/2.9
Observed reflections	200,313	331,361	250,327	262,154
Unique reflections	22,659	33,020	53,547	55,648
Space group	P2_1_2_1_2_1_	P2_1_2_1_2_1_	P2_1_	P2_1_
Unit cell dimensions (Å)				
a	50.405	50.445	57.932	57.968
b	56.430	56.664	40.977	40.906
c	113.759	113.920	68.416	68.387
alpha, beta, gamma (°)	90,90,90	90,90,90	90,110.70,90	90,110.68,90
Phasing statistics				
Number of selenium sites	6			
Contrast (SHELXE)	0.50			
RMS deviations				
bond lengths (Å)		0.027	0.007	0.007
bond angles (°)		2.03	2.18	1.11
Ramachandran plot (favored/allowed)		91.8/100	90.8/100	92.2/100
Mean B value for all atoms		16.14	11.95	13.34
R_cryst/_R_free_		15.7/20.0	16.7/19.3	16.7/19.0

**Figure 8:**
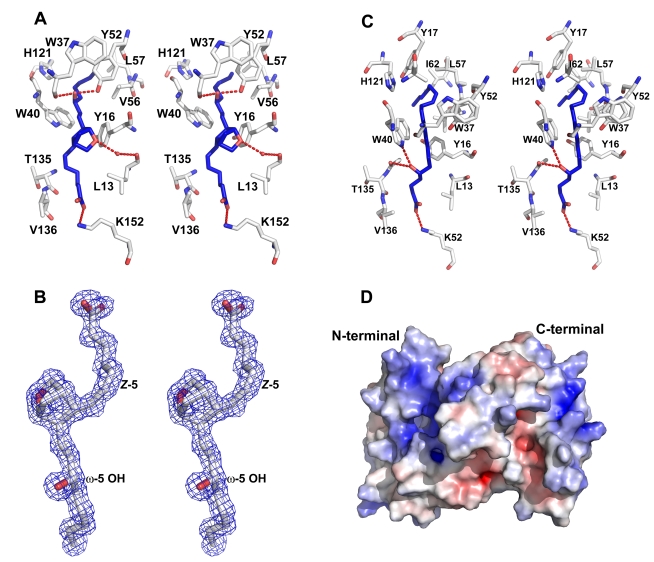
The AnSt-D7L1 N-terminal domain ligand binding site. (A) Stereoview of the binding interactions of U46619 at the ligand binding site. In the ligand, carbon atoms are colored blue and oxygen atoms red. Protein residues have carbon colored white, oxygen red, and nitrogen blue. Hydrogen bonds are shown as dashed red lines. (B) Stereoview of the 2Fo-Fc electron density map covering the U46619 ligand at the AnSt-D7L1 binding site contoured at 1.0 σ. In the ligand, carbon atoms are colored blue and oxygen atoms red. The “cis” unsaturation at position 5 and the hydroxyl group at the ω-5 position are labeled. (C) Stereoview of binding interactions of LTC_4_ in the AnSt-D7L1 N-terminal domain ligand binding pocket. Since the peptide moiety of the ligand is not visible in the crystal structure, only the fatty acid portion (5(S)-hydroxy- *(E,E,Z,Z)*-7, 9, 11, 14-eicosatetraenoic acid) of the molecule is shown. In the ligand, carbon atoms are shown in blue and oxygen atoms in red. Protein atoms are colored as in (A). Hydrogen bonds are shown as red dashed lines. (D) The solvent-accessible surface of AnSt-D7L1 is colored by electrostatic potential. The N- and C-terminal domains are labeled. Note the concentration of positive electrostatic potential surrounding the binding site for U46619 and CysLTs.

The N-terminal domain of AnSt-D7L1 consists of seven α-helices oriented around a solvent-accessible hydrophobic channel (Figure 7A,B). Superposition of the N-terminal domains of AeD7 and AnSt-D7L1 shows the two to be quite similar in structure, with an RMS positional deviation for 144 Cα positions of 1.36 Å (Figure S4). Like AeD7, the apparent entry point to the ligand-binding channel is surrounded by a positively charged electrostatic surface that would be attractive to the anionic eicosanoid ligands (Figure 8D) [15].

In AnSt-D7L1 the C-terminal domain also contains seven α-helices and is less similar in overall structure to AeD7 than is the N-terminal domain (Figures 7A,B and S4). The C-terminal domain of AeD7 is made up of seven α-helices in the ligand-free form and an eighth helix (H2) forms in response to the binding of norepinephrine (Figure 7C,D) [15]. This helix corresponds to the seventh α-helix (H2) of AnSt-D7L1 (Figure 7A–D) [15]. In AnSt-D7L1, the second α-helix of AeD7 (B2) is not present (Figure 7A,B). Rather, this region forms a predominantly coil structure extending between Lys 169 and Ser 184 containing a β-turn between residues Trp 173 and Gly 176. This portion of the C-terminal domain also lacks a cysteine residue found in AeD7 (Cys 173) and other D7 proteins, which forms a disulfide bond with a second cysteine (Cys 301 in AeD7) located at or near the C-terminal end of the protein (Figure 7C,D) [15],[16]. As a result of these changes, the terminal α-helix of the C-terminal domain (H2) is positioned differently than the corresponding α-helix H2 in AeD7.

**Figure 9:**
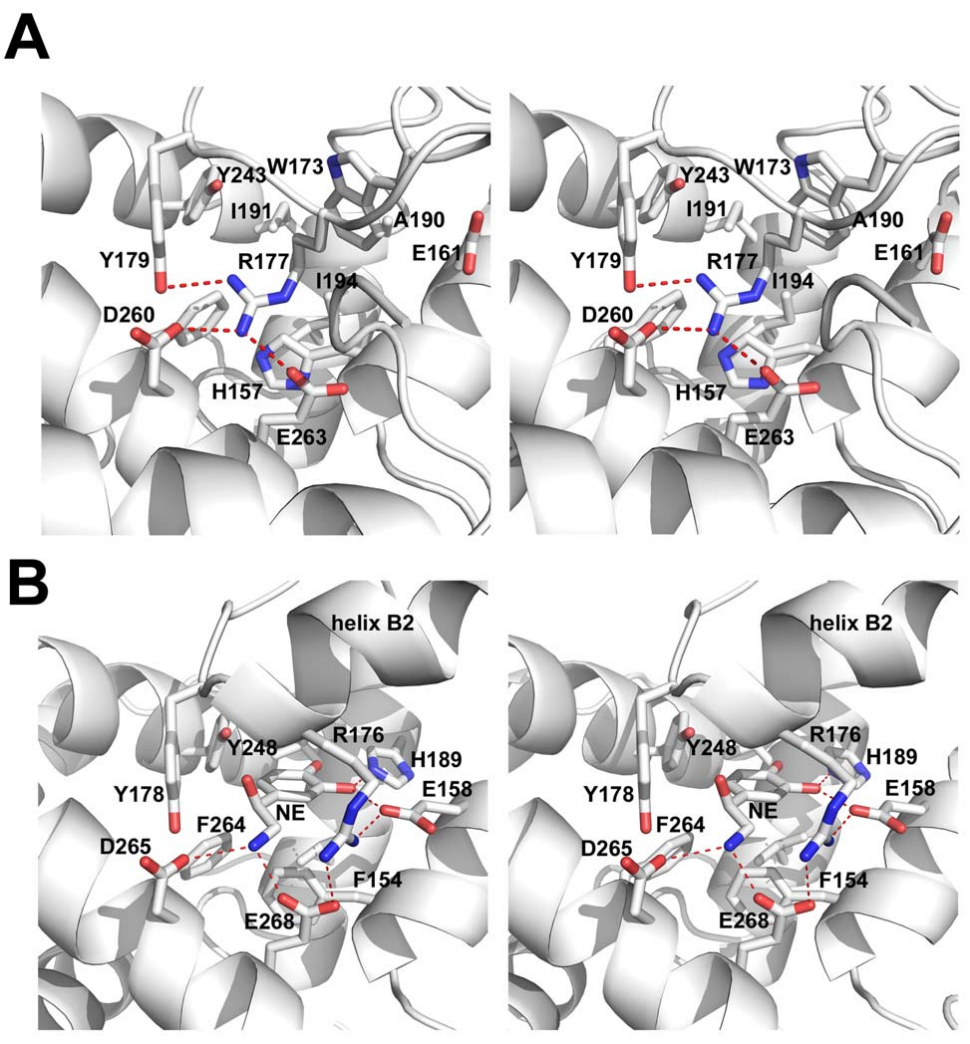
Detail of the C-terminal domain structure of AnSt-D7L1 and AeD7. (A) Stereoview of the region from the C-terminal domain of AnSt-D7L1 corresponding to the biogenic amine binding site of AeD7. Carbon atoms are colored in white, oxygen in red, nitrogen in blue. Helical segments are shown in ribbon format and hydrogen bonds are shown as dashed red lines. (B) Stereoview of the biogenic amine binding pocket (including a bound norepinephrine molecule labeled NE) of AeD7 shown from approximately the same view as AnSt-D7L1 in panel (A). Helical segment B2 is labeled to highlight how the conformational difference in this portion of the structure affects the positioning of residues in the ligand-binding pocket.

Many of the residues forming the C-terminal domain ligand binding pocket in AeD7 are conserved in AnSt-D7L1, but some occupy different positions, giving a completely different architecture to the structure of the region. Arg 177 in AnSt-D7L1 is equivalent to Arg 176 in AeD7 but is positioned such that its side chain partially occupies the same space as the ligand in the AeD7-norepinephrine complex (Figure 9A,B). Along with other changes, including the presence of tryptophan rather than leucine at position 173 in AnSt-D7L1, this modification acts to further fill the space equivalent to the biogenic amine binding pocket of the AeD7 C-terminal domain (Figure 9A,B). Although the identities of most of the residues forming hydrogen bonds with biogenic amines in AeD7 are conserved in AnSt-D7L1, some changes are apparent. Most notably, a histidine residue (position 189 in AeD7) that forms hydrogen bonds with the phenolic hydroxyl groups of serotonin and norepinephine is mutated to alanine (Ala 190) in AnSt-D7L1 (Figure 9A,B). These positional rearrangements and amino acid substitutions clearly explain the loss of the biogenic amine binding function in AnSt-D7L1 and indicate that the C-terminal domain in AnSt-D7L1 performs a different function than its counterpart AeD7. This novel function has not yet been identified.

### Ligand Complexes of AnSt-D7L1: Details of the N-Terminal Ligand Binding Pocket

Anst-D7L1 was crystallized in the presence of a 1.3-fold molar excess of either U46619 or LTC_4_ using otherwise identical crystallization conditions as for the ligand-free form. Both ligand complexes crystallized in the space group P2_1_ rather than P2_1_2_1_2_1_, with a single protein molecule in the asymmetric unit. Rather than binding in the separate domains of AnSt-D7L1, both ligands were found to bind in the hydrophobic pocket of the N-terminal domain of the protein.

The structure of the U46619 complex showed excellent electron density for the ligand, allowing a complete analysis of its binding interactions (Figure 8A,B). U46619 is bound with the unmodified end of the hydrocarbon chain projecting into the hydrophobic pocket, while the carboxyl group is positioned near the surface of the protein (Figure 8A). The hydroxyl group at the ω-5 position of the hydrocarbon chain of the ligand is stabilized by hydrogen bonds with the phenolic hydroxyl of Tyr 52 and the carbonyl oxygen of Trp 37, while the oxygen atom contained in the bicyclic ring system participates in a hydrogen bonding network with the carbonyl oxygen of Leu 13 via an intervening water molecule (Figure 8A). The carboxyl group of the ligand forms hydrogen bonds with the side chain of Lys 152, as well as with an ordered water molecule near the entry to the ligand binding channel, while the ω-end of the fatty acid chain sits in a hydrophobic pocket formed in part by the side chains of Leu 42, Val 56, and Leu 57 (Figure 8A).

The LTC_4_ complex of AnSt-D7L1 is similar in structure to the LTE_4_ complex of AeD7 with the ligand being bound at essentially the same site as U46619 (Figure 8C). The quality of the electron density for LTC_4_ was somewhat lower than for U46619, probably reflecting the relative instability of the ligand over the 2–3-week crystallization period. Nevertheless, the fatty acid portion of the molecule could be positioned in the ligand binding channel (Figure 8C). The peptide portion of LTC_4_ was not well ordered, consistent with the fact that similar thermodynamic parameters for the three CysLTs were observed in ITC experiments. The carboxyl group of the ligand is positioned similarly to U46619 and also forms a hydrogen bond or salt bridge with the side chain of Lys 152 (Figure 8C). The longer unsaturated hydrocarbon chain of LTC_4_ projects further into the ligand binding channel than does that of U46619. Near the position occupied by the ω-methyl group of U46619, the hydrocarbon chain of LTC_4_ is bent approximately 90° and the terminal four carbon atoms are accommodated in a hydrophobic cavity lined by the side chains of Tyr 117, Ile 62, Leu 60, Trp 40, and Leu 57 (Figure 8C).

The structure of the C-terminal domain in the ligand complexes is quite similar to the ligand-free protein and shows no evidence of interaction with either U46619 or LTC_4_. The position of helix H2 and the structure of the region corresponding to helix B2 in AeD7 are nearly identical to those seen in the ligand-free form of the protein. This again demonstrates that these differences between AnSt-D7L1 and AeD7 are not the result of crystal packing interactions. However, the region to the C-terminal side of helix H2 takes on a different conformation in the ligand complexes than in the ligand-free protein. In the absence of ligands, the segment between Leu 291 and the C-terminus (Phe 297) extends away from the surface of the protein and forms a β-turn between Thr 293 and His 296. In the ligand complexes the segment between Leu 291 and Val 295 packs against the surface of the protein, where it participates in a number of intramolecular hydrogen bonds (Figure S4). It is not clear whether the change in the C-terminal conformation is attributable to crystal packing differences or is a direct result of leukotriene binding in the N-terminal domain binding pocket.

## Discussion

Prevention of host hemostasis and inflammation is the primary function of the salivary secretion of blood feeding arthropods [2]–[4]. Toward this end an extremely diverse group of salivary proteins and peptides have evolved to inhibit key processes in these systems. Salivary protein genes tend to be highly duplicated and arranged in clusters. Often, individual members of these clusters have diverged in sequence and added new functions important for blood feeding. The duplication and divergence paradigm is well illustrated by the D7 salivary protein family in hematophagous Diptera, with members of this group playing numerous important roles in blood feeding. Isawa and co-workers described a single-domain D7 protein from *An. stephensi*, referred to as hamadarin, and found it to bind coagulation factor XII and high molecular weight kininogen [23]. This protein acts both to inhibit the contact pathway of coagulation and the generation of the proinflammatory peptide bradykinin. In *An. gambiae*, single-domain D7 proteins (D7r1, D7r2, D7r3, and D7r4) as well as the C-terminal domain of the two-domain D7 present in *Ae. aegypti* (AeD7) inhibit inflammation and vascular tone by binding biogenic amines [14]. One of these, D7r1, is an ortholog of hamadarin and has been shown to inhibit coagulation [14]. Calvo et al. [15] found that the N-terminal domain of AeD7 bound CysLTs, a group of proinflammatory lipid mediators, while the C-terminal domain bound biogenic amines in a manner similar to the single domain proteins. Here we have shown that the two-domain D7 protein from *An. stephensi*, AnSt-D7L1, binds CysLTs like AeD7 but also functions as an inhibitor of platelet activation by binding TXA_2_. AeD7 showed no detectable binding with the TXA_2_ analog U46619, while AnSt-D7L1 binds this ligand with high affinity. Additionally, the C-terminal domain has lost the ability to bind biogenic amines, a function found in apparent orthologs of this protein in culicine mosquitoes.

### The N-Terminal Domain and Eicosanoid Binding

The binding site for eicosanoid ligands lies in a pocket in the N-terminal domain of AnSt-D7L1, which is lined with mostly hydrophobic residues that accommodate a large portion of the fatty acid chain. CysLTs are substituted at the ω-14 position of the chain while TXA_2_ and its analogs contain a hydroxyl group at the ω-5 position (Figure S1). Binding of TXA_2_ analogs by AnSt-D7L1 appears to result from its ability to accommodate this substitution pattern. AnSt-D7L1 contains a tyrosine residue at position 52, while AeD7 has phenylalanine at the equivalent position. Consequently, AeD7 is unable to form one of the hydrogen bonds that stabilize U46619 in the AnSt-D7L1 binding pocket and does not bind TXA_2_ analogs [15]. The PGH_2_ analog U51605 is very similar in structure to U46619 but lacks the ω-5 hydroxyl group and binds with approximately 10-fold lower affinity to AnSt-D7L1, further demonstrating the importance of this functionality in binding. The sequences of the N-terminal domains of two-domain D7 proteins from all mosquitoes show a high degree of sequence conservation, particularly with regard to residues lining the binding pocket that are involved with ligand binding. Tyrosine-phenylalanine polymorphism at the equivalent of AnSt-D7L1 position 52 is found in both anopheline and culicine D7s. This suggests that mosquito salivary secretions contain D7 protein forms that bind both CysLTs and TXA_2_, as well as forms that bind only CysLTs, and that there may be an adaptive significance to differences in TXA_2_ binding observed in AeD7 and AnSt-D7L1.

### Functional Significance of AnSt-D7L1 Ligand Binding

Limitation of mast cell responses in host skin is essential for successful blood feeding, and scavengers of small-molecule mediators are apparently present in saliva to accomplish this task. Immediate cutaneous reactions to mosquito bites lead to release of histamine and LTC_4_, potent mediators of acute allergic processes and inflammation [36]. Leukotrienes are produced by activated mast cells, stimulated leukocytes, endothelial cells, and epithelial cells through conversion of AA to 5-HPETE catalyzed by 5-lipoxygenase [24],[37],[38]. The most physiologically active leukotrienes are LTB_4_ and the CysLTs. LTC_4_, the primary CysLT product, is secreted by cells and converted extracellularly to LTD_4_ and LTE_4_ by successive cleavages of its glutathione moiety.

In human skin it has been demonstrated that intradermal injection of nanomolar amounts of LTC_4_, LTD_4_, and LTE_4_ are able to elicit erythema and wheal formation [36],[39]. Although CysLTs promote smooth muscle contraction [24],[26] and consequent vasoconstriction in other tissues such as the pulmonary vasculature [25], they elicit a vasodilatatory response in human skin, increasing blood flow in a manner similar to histamine [40],[41]. Moreover, CysLTs, as well as histamine, cause increased vascular permeability and interstitial transport in the skin, favoring edema formation [36],[42].

The CysLT receptors, CysLT_1_ and CysLT_2_, are distributed in numerous tissues. In some endothelial populations, CysLT_1_ is much more abundant than CysLT_2_ and has affinities for LTD_4_ and LTC_4_ of approximately 1 nM and 10 nM, respectively. CysLT_2_ binds both LTC_4_ and LTD_4_ with an affinity of approximately 10 nM, and both receptors bind LTE_4_ with affinities near 100 nM [24]. The affinities (ranging from ∼4 to 6 nM) of AnSt-D7L1 for all the three CysLTs are similar to one another and are equal to or lower than those described for CysLT receptors, making it likely that AnSt-D7L1 can compete with CysLT receptors for ligands if present at sufficient concentration.

Unlike the previously described AeD7, AnSt-D7L1 also binds TXA_2_ analogs, and evidence from platelet aggregation assays strongly suggests that it binds TXA_2_ itself. Besides being a potent platelet activation agonist, TXA_2_ induces smooth muscle contraction in vascular tissue [27], and like the CysLTs is able to cause itching [43]. Therefore, TXA_2_ interferes with mosquito feeding by promoting hemostasis (vasoconstriction and platelet aggregation), as well as by causing inflammatory skin responses.

Mosquito bites cause injury to the skin, leading to the exposure of collagen present in subendothelial extracellular matrix [44]. Exposed collagen interacts directly or indirectly (via VWF) with different receptors present on the platelet surface triggering platelet activation, aggregation, and thrombus formation. Among these receptors is GPVI, a central collagen receptor that is able to bind collagen both in high and low shear conditions, promoting inside out activation of integrin receptors α_2_β_1_ and α_IIb_β_3_, thereby increasing the affinities for their respective ligands, resulting in aggregation of platelets. Activated platelets release granules containing small molecule agonists such as ADP and begin synthesizing TXA_2_. These secondary mediators are able to diffuse and activate circulating platelets that do not have contact with collagen, resulting in potentiation of the pro-aggregatory signal and an increase in the size of the platelet plug. Studies comparing the effects of collagen with other GPVI agonists, such as collagen-related peptide (CRP) [45], and platelets lacking protein G_q_ have shown that ADP and TXA_2_ are essential for collagen-induced platelet aggregation.

The effect of AnSt-D7L1 on collagen-induced aggregation is similar to that obtained when platelets are treated with indomethacin or SQ 29,548, strongly suggesting that this protein is able to scavenge the TXA_2_ generated in response to collagen exposure. Moreover, similar effects have been shown in previous studies where platelets lacking G_αq_ and G_α13_, G proteins coupled to TP and to ADP receptors, were exposed to collagen [46].

Binding of TXA_2_ or its analogs to TP receptors on the platelet surface activates the platelet, leading to shape change followed by aggregation [27]. When the analog U46619 is used to promote aggregation, the presence of AnSt-D7L1 at near equimolar concentrations is enough to completely inhibit the platelet aggregation response. AnSt-D7L1 also abrogates AA-induced aggregation, strongly suggesting that it scavenges TXA_2_ being produced as a result of stimulation by its biosynthetic precursor. A recently described mutation in the human TP receptor causes platelet dysfunction and lack of responsiveness to AA and U46619 [47] at similar doses as used in the present work and has similar effects as those described here for AnSt-D7L1.

In order for this binding mechanism to be effective in vivo, the protein concentration must be sufficient to remove a large fraction of the eicosanoid ligand from the feeding site. Calvo et al. [14] have estimated the quantities of a biogenic amine-binding protein that is necessary to accomplish this task to be 0.03–0.3 µg per feeding site. Mosquito salivary glands contain approximately 1–3 µg of protein, and a mosquito injects about half of this protein during a single feeding. Injection of 1 nmol of CysLT at a concentration of 50 µM into a small site on the skin has been shown to result in the appearance of large erythemas measuring over 20 mm in diameter and persisting for many hours [36]. The far less severe response to mosquito feeding suggests that the CysLT concentrations at the feeding site would be much lower than this. Likewise, the data presented here indicate that a concentration of AnSt-D7L1 of 0.03 µM is sufficient to remove a significant fraction of the TXA_2_ secreted by platelets in response to collagen stimulation and that 0.1 µM eliminates most of the aggregation response (Figure 5). This would suggest that the TXA_2_ concentration in uniformly activated platelet-rich plasma is near 0.1 µM. Based on the size of a blood meal, Calvo et al. [14] estimate the feeding volume of a mosquito to be maximally 10 µL, and considering the relative concentration of two-domain D7 proteins in the salivary secretion of *An. gambiae* [48], it appears that approximately 5–10 ng of these two-domain proteins are injected during a single feeding period, giving a final concentration of approximately 0.1 µM in the feeding volume.

### The C-Terminal Domain and D7 Evolution

The highly expressed single-domain D7 proteins in *Anopheles* species are known to bind biogenic amine ligands including serotonin, norepinephrine, and histamine. Comparisons of these sequences with the C-terminal domains of the two-domain D7s from culicine species show a high degree of sequence conservation of the residues directly involved with ligand binding (Figure 1; [15]) The crystal structures of AeD7 (two-domain) from *Ae. aegypti* and D7r4 (one-domain) from *An. gambiae* also show very similar binding modes for biogenic amine ligands, with most hydrogen bonding interactions being highly conserved between the two. Phylogenetic analyses of D7 sequences suggest that the two-domain forms are ancestral and that the single domain proteins found in both culicine and anopheline mosquitoes are derived from an apparent duplication of the C-terminal domain of a two-domain D7 protein [14]. In culicine mosquitoes, the function of the single-domain proteins is not known, but a lack of conservation of residues involved with biogenic amine binding suggests that biogenic amines are not the target ligand [16].

Sequence comparisons of apparently orthologous two-domain D7s from culicine and anopheline species initially suggested that the C-terminal domain of the anopheline proteins lack some important determinants of biogenic amine binding, while maintaining a high overall degree of similarity with the culicine forms. The work presented here shows that the biogenic amine binding function has indeed been lost in the anopheline proteins and that the loss of function is due to rearrangements that dramatically affect the structure of the binding pocket. The loss of α-helix B2 that is found in both AeD7 and D7r results in Arg 177 taking on a different conformation in AnSt-D7L1. Along with the bulky side chain of Trp 173, Arg 177 fills most of what would be the biogenic amine binding pocket in the D7 forms that bind these ligands (Figure 9A,B). Two crystal forms of AnSt-D7L1 are described in this study. The orthorhombic form is obtained in the absence of eicosanoid ligands, while the monoclinic form is obtained in their presence. In the two forms, the structure of the C-terminal domain binding pocket region is nearly identical (Figure S4), indicating that interactions in the crystal play no role in producing these differences between AnSt-D7L1 and AeD7.

It appears that the cluster of single-domain D7 proteins in anopheline mosquitoes has taken over the function of the C-terminal domain of the two-domain D7s of culicine (*Aedes* and *Culex*) mosquitoes, making it unnecessary for AnSt-D7L1 and other anopheline forms to maintain the biogenic amine-binding function. Apparently, another function has been acquired by the C-terminal domain of these proteins that may be entirely unrelated to the binding of small-molecule ligands. This case seems to be an example of extensive duplication of salivary protein genes allowing structural and functional diversification within protein families. The D7 protein family represents a case where a duplication of one domain of an ancestral two-domain protein to form a one-domain form has allowed the ancestral protein to lose its original function and almost certainly acquire a new one. We are currently working to determine the more recently acquired function of the C-terminal domain in this group of proteins.

## Materials and Methods

### Materials

UltraPure guanidinium hydrochloride was obtained from Invitrogen. L-arginine, serotonin, norepinephrine, histamine, ADP, PMA, and indomethacin were obtained from Sigma-Aldrich. All leukotrienes and prostaglandins as well as U46619, carboxyclic TXA_2_, TXB_2_, U51605, AA (for ITC experiment), PAF, and SQ 29,548 were obtained from Cayman Chemical. Native collagen fibrils (type I) from equine tendons, AA, and ristocetin used in platelet experiments were purchased from Chrono-Log Corporation. Convulxin and RPAI1 were prepared as described previously [22],[49].

### Protein Expression, Refolding, and Purification

The AnSt-D7L1 cDNA, minus its signal sequence, was cloned into the expression vector pET17b and was expressed in BL21(DE3)pLysS cells grown for 3 h after induction with 1 mM IPTG [50]. After expression, inclusion bodies were washed in Triton X-100 as previously reported [50] and denatured in 100 mL of 6 M guanidinium HCl and 20 mM Tris-HCl pH 8.0 containing 10 mM DTT. This solution (50 mL) was refolded by adding dropwise to 4 L of 0.3 M L-arginine in 50 mM CAPS buffer pH 10.0. The solution was stirred for 1 h at 25°C and transferred to 4°C overnight. The protein was concentrated and purified by gel filtration chromatography on Sephacryl S-100, followed by anion exchange chromatography on SP sepharose. The protein was further purified by a final step of gel filtration chromatography on Superdex 75. Purity was assessed by SDS-PAGE (Figure S5).

### Isothermal Titration Calorimetry (ITC)

AnSt-D7L1 was prepared for ITC experiments by dialysis against 20 mM Tris-HCl 0.15 M NaCl pH 7.4 for 1 h. Binding experiments were performed on a MicroCal VP-ITC instrument. Aliquots of lipid stock solutions in ethanol or methyl acetate were dried under a stream of nitrogen in a glass vial, then dissolved in 20 mM Tris HCl, 0.15 M NaCl pH 7.4, vortexed, and sonicated for 8 min. Non-lipidic ligands were dissolved in 20 mM Tris HCl, 0.15 M NaCl pH 7.4. All solutions were degassed prior to use. Assays were performed at 35°C with successive 10 µL injections, with a spacing interval of 180 s and a syringe stirring speed of 290 rpm. In all the cases, after subtraction of the heats of dilution, the measured heats were converted to enthalpies and analyzed by fitting to a single-site binding model using the Microcal Origin software.

### Smooth Muscle Bioassays

Guinea pig ileum contraction in response to LTC_4_ was measured isotonically with an initial load of 1.0 g tension, and rat aorta ring contraction in response to U46619 was measured isometrically after a distension of 2.5 g. Tissue preparations were bathed in modified Tyrode solution (with 10 mM HEPES buffer, pH 7.4) during the course of the experiment, and the solutions were kept oxygenated by bubbling air into the bath [51].

### Platelet Preparation and Platelet Aggregation Assay

Platelets were prepared as described previously [52]. Human platelet-rich plasma (∼2×10^5^/µL final density) and Tyrode-BSA buffer [52] in the presence or absence of AnSt-D7L1 (final volume 300 µL) were placed in an aggregometer [52] and stirred at 1,200 rpm at 37°C for 1 min prior to the addition of different agonists. In experiments using the human TP (TXA_2_ receptor) antagonist SQ 29,548 or made in the presence of proteins (AnSt-D7L1 or RPAI-1), those compounds were added to the platelet suspension and Tyrode mix prior to addition of agonists. In experiments in which indomethacin, a general cycloxygenase inhibitor, was used, platelets were pre-incubated with the compound for 5 min prior to addition of agonists.

### Crystallization and Data Collection

AnSt-D7L1 was crystallized using the hanging drop vapor diffusion method from 0.1 M Tris pH 8.5, 0.2 M lithium sulfate, 30% PEG 3000. The crystals were frozen for data collection in the crystallization buffer described above, containing 10% glycerol. To prepare a selenomethionine derivative of AnSt-D7L1, the expression plasmid construct was transformed into BL834(DE3)pLysS cells and grown in SelenoMet media (Molecular Dimensions Ltd.) as per the manufacturer's instructions. In order to obtain crystals with the protein bound to its ligands, either LTC_4_ or U46619 prepared in 10 mM Tris-HCl pH 8.0 buffer was pre-incubated with AnSt-D7L1 for 10 min but in a 1.3 molar excess when compared to the protein concentration. Prior to data collection, the crystals were frozen using the cryoprotectant described above.

Data collection was performed at beamlines 22-BM at the Southeast Regional Collaborative Access Team (SER-CAT) and 19-BM of the Structural Biology Center (SBC), Advanced Photon Source (APS), Argonne National Laboratory. Crystals of the ligand-free form of the protein belong to the space group P2_1_2_1_2_1_ and contain one monomer per asymmetric unit (Table 1). Crystals of the LTC_4_ and U46619 complexes belong to the space group P2_1_ and also contain one monomer per asymmetric unit. Four data sets were collected from a ligand-free selenomethionine crystal (2.00 Å resolution), a ligand-free crystal (1.77 Å resolution), a U46619-complexed co-crystal (1.45 Å resolution), and a LTC_4_-complexed co-crystal (1.43 Å resolution).

### Structure Solution and Refinement

The structure of AnSt-D7L1 was determined using a combination of single wavelength anomalous dispersion (SAD) methods with data collected at the selenium absorption edge, and molecular replacement using the N-terminal domain of AeD7 from *Ae. aegypti* as the search model (Table 1) [15]. The data were indexed, integrated, and scaled using HKL2000 or HKL3000 [53]. Initial phases were obtained from the selenium data using SHELX-C, D, and E [54],[55]. After molecular replacement using PHASER [56] with the selenomethionine data set, a model of the N-terminal domain of AnSt-D7L1 was refined using REFMAC [57] including the phase information from the SAD experiment. After this procedure, electron density for some of the helical segments in the C-terminal domain could be observed, allowing manual building of this portion of the model in Coot [58]. The complete model was refined using REFMAC [57], with a TLS model being applied in the later stages utilizing a single TLS group [57]. The structures of the ligand complexes were determined by molecular replacement using PHASER, with the ligand-free protein as a search model [59]. The structures were rebuilt using Coot and refined using REFMAC. Since little electron density was observed for the peptidyl portion of LTC_4_, the ligand was modeled as only its lipid portion, 5(S)-hydroxy-*(E,E,Z,Z)*-7,9,11,14-eicosatetraenoic acid. The structures of ligand-free AnSt-D7L1 (3NGV), its U46619 complex (3NHT), and its LTC_4_ complex (3NHI) have been deposited with the RCSB protein data bank.

## Supporting Information

Figure S1 **Structures of leukotrienes and thromboxanes and thromboxane analogs.** (A) LTC_4_, (B) LTD_4_, (C) LTE_4_, (D) TXA_2_, (E) carbocyclic TXA_2_, (F) U46619, (G) U51605.
(1.67 MB TIF)

Figure S2 **Gel filtration of the AnSt-D7L1-LTC_4_ complex (A) and free LTC_4_ (B) on a Superdex peptide column.** (A) AnSt-D7L1 (100 µL) 10 µM and LTC_4_ 10 µM were incubated together to form a complex which was then injected onto the column. (B) LTC_4_ was injected alone in order to obtain a retention volume measurement for the free ligand. In both cases, the column was eluted with 100 mM ammonium acetate pH 7.2 at a flow rate of 60 µL per min.
(0.39 MB TIF)

Figure S3 **Mass spectral analysis of the AnSt-D7L1-LTC_4_ complex after gel filtration chromatography.** Top: The peak fraction from the gel filtration of Figure S2 (3 µl) was applied to a C18 column and eluted with 5% methanol 0.1% acetic acid for 10 min, then 5%–95% methanol 0.1% acetic acid (0.3 mm diameter column in 20 min at 3 µl/min). The eluent was analyzed directly by MS/MS. Bottom is the same analysis applied to 1 µl of 10 µM LTC_4_ solution. The chromatograms represent abundance of the m/z 626.7 molecular ion (M+H) of LTC_4_. The inset is a sample collision-induced dissociation spectrum (35% intensity) recorded at 61–65 min.
(2.15 MB TIF)

Figure S4 **Structural comparisons of D7 proteins.** (A) Stereoview of superposed carbon alpha traces of the C-terminal domains of AnSt-D7L1 (red) and AeD7 (black). Helix B2 of AeD7 and H2 from both proteins are labeled in the appropriate colors. (B) Stereoview of superposed N-terminal domains from ligand-free AnSt-D7L1 and its U46619 complex. The N-termini are labeled. (C) Stereoview of superposed C-terminal domains from ligand-free AnSt-D7L1 and its U46619 complex. The difference in position of the C-terminus for each protein is indicated in the appropriate color.
(2.52 MB TIF)

Figure S5 **Purification of recombinant AnSt-D7L1.** (A) Gel filtration chromatography of purified AnSt-D7L1 on Superdex 75. Elution buffer: 20 mM Tris HCl pH 8.0, 0.15 M NaCl. (B) SDS-PAGE gel of purified recombinant AnSt-D7L1 stained with Coomassie blue.
(5.20 MB TIF)

## Abbreviations

AA: arachidonic acid
ADP adenosine diphosphate
CysLT: cysteinyl leukotriene
ITC: isothermal titration calorimetry
LTB_4_: leukotriene B_4_
LTC_4_: leukotriene C_4_
LTD4: leukotriene D_4_
LTE_4_: leukotriene E_4_
OBP: odorant-binding protein
PAF: platelet activating factor
PE: phenylephrine
PGD_2_: prostaglandin
PGE_2_: prostaglandin E_2_
PGF_2a_: prostaglandin F_2α_
PGH_2_: prostaglandin H_2_
PMA: phorbol-12-myristate-13-acetate
RMS: root-mean-square
TXA_2_: thromboxane
TXB_2_: thromboxane B_2_
VWF: von Willibrand factor

## References

[1] Ribeiro J. M (1995). Blood-feeding arthropods: live syringes or invertebrate pharmacologists?. Infect Agents Dis.

[2] Ribeiro J. M, Francischetti I. M (2003). Role of arthropod saliva in blood feeding: sialome and post-sialome perspectives.. Annu Rev Entomol.

[3] Champagne D. E (2005). Antihemostatic molecules from saliva of blood-feeding arthropods.. Pathophysiol Haemost Thromb.

[4] Valenzuela J. G (2002). High-throughput approaches to study salivary proteins and genes from vectors of disease.. Insect Biochem Mol Biol.

[5] Titus R. G, Ribeiro J. M (1988). Salivary gland lysates from the sand fly Lutzomyia longipalpis enhance Leishmania infectivity.. Science.

[6] Valenzuela J. G, Belkaid Y, Garfield M. K, Mendez S, Kamhawi S (2001). Toward a defined anti-Leishmania vaccine targeting vector antigens: characterization of a protective salivary protein.. J Exp Med.

[7] de Moura T. R, Oliveira F, Novais F. O, Miranda J. C, Clarencio J (2007). Enhanced Leishmania braziliensis infection following pre-exposure to sandfly saliva.. PLoS Negl Trop Dis.

[8] Donovan M. J, Messmore A. S, Scrafford D. A, Sacks D. L, Kamhawi S (2007). Uninfected mosquito bites confer protection against infection with malaria parasites.. Infect Immun.

[9] Edwards J. F, Higgs S, Beaty B. J (1998). Mosquito feeding-induced enhancement of Cache Valley Virus (Bunyaviridae) infection in mice.. J Med Entomol.

[10] Limesand K. H, Higgs S, Pearson L. D, Beaty B. J (2000). Potentiation of vesicular stomatitis New Jersey virus infection in mice by mosquito saliva.. Parasite Immunol.

[11] Sutherland G. B, Ewen A. B (1974). Fecundity decrease in mosquitoes ingesting blood from specifically sensitized mammals.. J Insect Physiol.

[12] Francischetti I. M (2009). Platelet aggregation inhibitors from hematophagous animals.. Toxicon, in press.

[13] Andersen J. F (2009). Structure and mechanism in salivary proteins from blood-feeding arthropods.. Toxicon, in press.

[14] Calvo E, Mans B. J, Andersen J. F, Ribeiro J. M (2006). Function and evolution of a mosquito salivary protein family.. J Biol Chem.

[15] Calvo E, Mans B. J, Ribeiro J. M, Andersen J. F (2009). Multifunctionality and mechanism of ligand binding in a mosquito antiinflammatory protein.. Proc Natl Acad Sci U S A.

[16] Mans B. J, Calvo E, Ribeiro J. M, Andersen J. F (2007). The crystal structure of D7r4, a salivary biogenic amine-binding protein from the malaria mosquito Anopheles gambiae.. J Biol Chem.

[17] Mans B. J, Ribeiro J. M (2008). A novel clade of cysteinyl leukotriene scavengers in soft ticks.. Insect Biochem Mol Biol.

[18] Mans B. J, Ribeiro J. M, Andersen J. F (2008). Structure, function, and evolution of biogenic amine-binding proteins in soft ticks.. J Biol Chem.

[19] Ribeiro J. M, Walker F. A (1994). High affinity histamine-binding and antihistaminic activity of the salivary nitric oxide-carrying heme protein (nitrophorin) of Rhodnius prolixus.. J Exp Med.

[20] Andersen J. F, Gudderra N. P, Francischetti I. M, Ribeiro J. M (2005). The role of salivary lipocalins in blood feeding by Rhodnius prolixus.. Arch Insect Biochem Physiol.

[21] Ribeiro J. M, Arca B (2009). From Sialomes to the Sialoverse: an insight into salivary potion of blood-feeding insects..

[22] Francischetti I. M, Ribeiro J. M, Champagne D, Andersen J (2000). Purification, cloning, expression, and mechanism of action of a novel platelet aggregation inhibitor from the salivary gland of the blood-sucking bug, Rhodnius prolixus.. J Biol Chem.

[23] Isawa H, Yuda M, Orito Y, Chinzei Y (2002). A mosquito salivary protein inhibits activation of the plasma contact system by binding to factor XII and high molecular weight kininogen.. J Biol Chem.

[24] Boyce J. A (2005). Eicosanoid mediators of mast cells: receptors, regulation of synthesis, and pathobiologic implications.. Chem Immunol Allergy.

[25] Hanna C. J, Bach M. K, Pare P. D, Schellenberg R. R (1981). Slow-reacting substances (leukotrienes) contract human airway and pulmonary vascular smooth muscle in vitro.. Nature.

[26] Findlay S. R, Lichtenstein L. M, Siegel H, Triggle D. J (1981). Mechanisms of contraction induced by partially purified slow reacting substance from human polymorphonuclear leukocytes and leukotriene D in guinea pig ileal smooth muscle.. J Immunol.

[27] Nakahata N (2008). Thromboxane A2: physiology/pathophysiology, cellular signal transduction and pharmacology.. Pharmacol Ther.

[28] Andrews R. K, Berndt M. C (2004). Platelet physiology and thrombosis.. Thromb Res.

[29] Canobbio I, Balduini C, Torti M (2004). Signalling through the platelet glycoprotein Ib-V-IX complex.. Cell Signal.

[30] Clemetson K. J, Clemetson J. M (2001). Platelet collagen receptors.. Thromb Haemost.

[31] Nieswandt B, Watson S. P (2003). Platelet-collagen interaction: is GPVI the central receptor?. Blood.

[32] Surin W. R, Barthwal M. K, Dikshit M (2008). Platelet collagen receptors, signaling and antagonism: emerging approaches for the prevention of intravascular thrombosis.. Thromb Res.

[33] Kahner B. N, Shankar H, Murugappan S, Prasad G. L, Kunapuli S. P (2006). Nucleotide receptor signaling in platelets.. J Thromb Haemost.

[34] Francischetti I. M, Andersen J. F, Ribeiro J. M (2002). Biochemical and functional characterization of recombinant Rhodnius prolixus platelet aggregation inhibitor 1 as a novel lipocalin with high affinity for adenosine diphosphate and other adenine nucleotides.. Biochemistry.

[35] Ohkubo S, Nakahata N, Ohizumi Y (1996). Thromboxane A2-mediated shape change: independent of Gq-phospholipase C—Ca2+ pathway in rabbit platelets.. Br J Pharmacol.

[36] Soter N. A, Lewis R. A, Corey E. J, Austen K. F (1983). Local effects of synthetic leukotrienes (LTC4, LTD4, LTE4, and LTB4) in human skin.. J Invest Dermatol.

[37] Boyce J. A (2007). Mast cells and eicosanoid mediators: a system of reciprocal paracrine and autocrine regulation.. Immunol Rev.

[38] Murphy R. C, Gijon M. A (2007). Biosynthesis and metabolism of leukotrienes.. Biochem J.

[39] Camp R. D, Coutts A. A, Greaves M. W, Kay A. B, Walport M. J (1983). Responses of human skin to intradermal injection of leukotrienes C4, D4 and B4.. Br J Pharmacol.

[40] Bisgaard H (1987). Vascular effects of leukotriene D4 in human skin.. J Invest Dermatol.

[41] Bisgaard H, Kristensen J, Sondergaard J (1982). The effect of leukotriene C4 and D4 on cutaneous blood flow in humans.. Prostaglandins.

[42] Bisgaard H, Lerche A, Kristensen J. K (1985). Leukotriene- and histamine-induced increases in vascular permeability and interstitial transport in the skin.. J Invest Dermatol.

[43] Andoh T, Nishikawa Y, Yamaguchi-Miyamoto T, Nojima H, Narumiya S (2007). Thromboxane A2 induces itch-associated responses through TP receptors in the skin in mice.. J Invest Dermatol.

[44] Watson S. P (2009). Platelet activation by extracellular matrix proteins in haemostasis and thrombosis.. Curr Pharm Des.

[45] Jarvis G. E, Atkinson B. T, Snell D. C, Watson S. P (2002). Distinct roles of GPVI and integrin alpha(2)beta(1) in platelet shape change and aggregation induced by different collagens.. Br J Pharmacol.

[46] Moers A, Wettschureck N, Gruner S, Nieswandt B, Offermanns S (2004). Unresponsiveness of platelets lacking both Galpha(q) and Galpha(13). Implications for collagen-induced platelet activation.. J Biol Chem.

[47] Mumford A. D, Dawood B. B, Daly M. E, Murden S. L, Williams M. D A novel thromboxane A2 receptor D304N variant that abrogates ligand binding in a patient with a bleeding diathesis.. Blood.

[48] Choumet V, Carmi-Leroy A, Laurent C, Lenormand P, Rousselle J. C (2007). The salivary glands and saliva of Anopheles gambiae as an essential step in the Plasmodium life cycle: a global proteomic study.. Proteomics.

[49] Francischetti I. M, Saliou B, Leduc M, Carlini C. R, Hatmi M (1997). Convulxin, a potent platelet-aggregating protein from Crotalus durissus terrificus venom, specifically binds to platelets.. Toxicon.

[50] Andersen J. F, Francischetti I. M, Valenzuela J. G, Schuck P, Ribeiro J. M (2003). Inhibition of hemostasis by a high affinity biogenic amine-binding protein from the saliva of a blood-feeding insect.. J Biol Chem.

[51] Webster M. E, Prado E. S, Perlman G. E, Lorand L (1970). Glandular kallikreins from horse and human urine and from hog pancreas.. Methods in enzymology.

[52] Lagrue A. H, Francischetti I. M, Guimaraes J. A, Jandrot-Perrus M (1999). Phosphatidylinositol 3′-kinase and tyrosine-phosphatase activation positively modulate Convulxin-induced platelet activation. Comparison with collagen.. FEBS Lett.

[53] Minor W, Cymborowski M, Otwinowski Z, Chruszcz M (2006). HKL-3000: the integration of data reduction and structure solution—from diffraction images to an initial model in minutes.. Acta Crystallogr D Biol Crystallogr.

[54] Schneider T. R, Sheldrick G. M (2002). Substructure solution with SHELXD.. Acta Crystallogr D Biol Crystallogr.

[55] Sheldrick G. M (2002). Macromolecular phasing with SHELXE.. Z Kristallogr.

[56] McCoy A. J (2007). Solving structures of protein complexes by molecular replacement with Phaser.. Acta Crystallogr D Biol Crystallogr.

[57] CCP4 (1994). The CCP4 suite: programs for protein crystallography.. Acta Crystallogr D Biol Crystallogr.

[58] Emsley P, Cowtan K (2004). Coot: model-building tools for molecular graphics.. Acta Crystallogr D Biol Crystallogr.

[59] Kleywegt G. J, Jones T. A (1995). Where freedom is given, liberties are taken.. Structure.

