# Supplementary figures and images for: The Function and Three-Dimensional Structure of a Thromboxane A2/Cysteinyl Leukotriene-Binding Protein from the Saliva of a Mosquito Vector of the Malaria Parasite
Source: PLoS Biol. 2010 Nov 30;8(11):e1000547. doi: 10.1371/journal.pbio.1000547 (PMC2994686; doi:10.1371/journal.pbio.1000547)

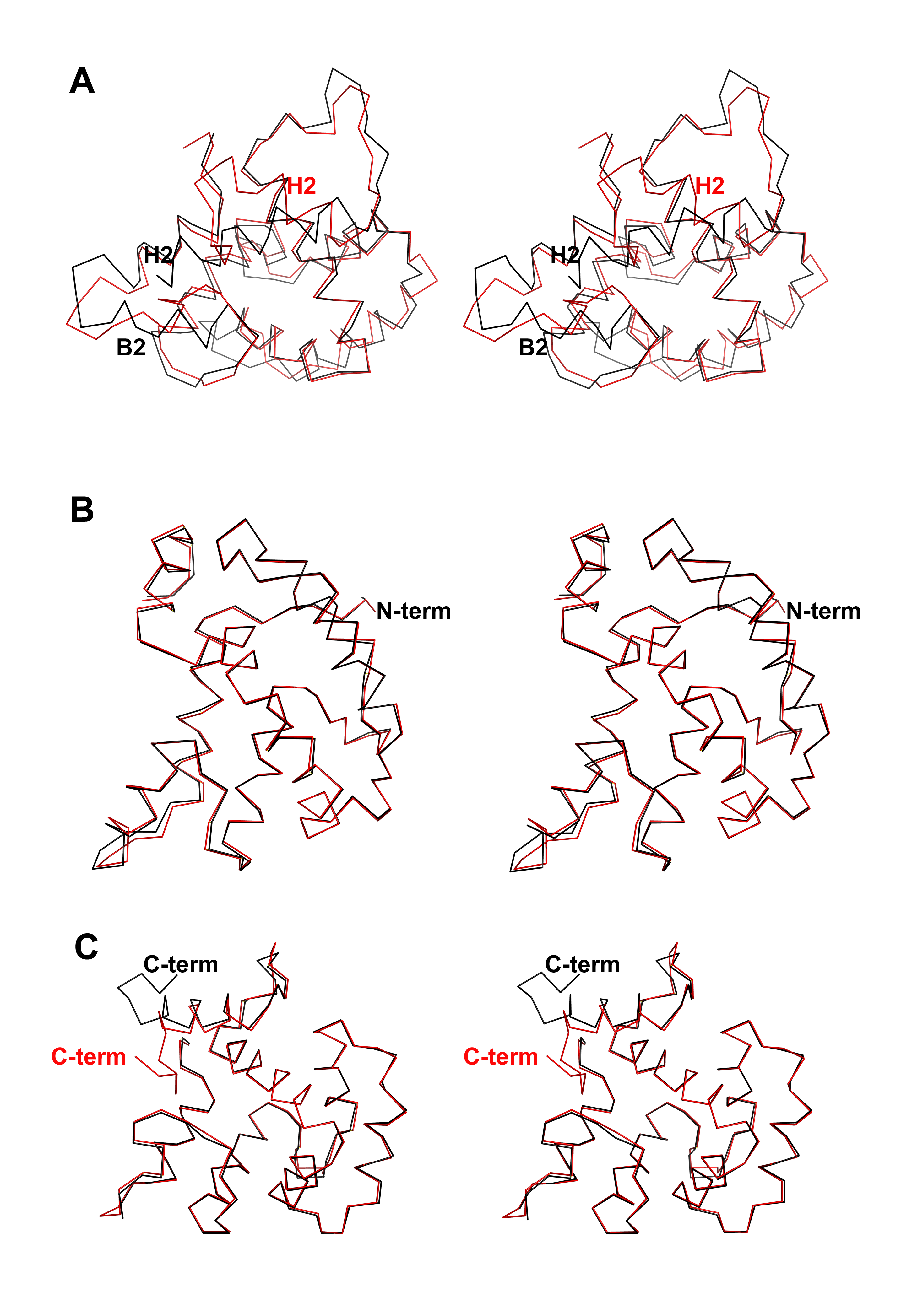

Supplement: Figure S4 — Structural comparisons of D7 proteins. (A) Stereoview of superposed carbon alpha traces of the C-terminal domains of AnSt-D7L1 (red) and AeD7 (black). Helix B2 of AeD7 and H2 from both proteins are labeled in the appropriate colors. (B) Stereoview of superposed N-terminal domains from ligand-free AnSt-D7L1 and its U46619 complex. The N-termini are labeled. (C) Stereoview of superposed C-terminal domains from ligand-free AnSt-D7L1 and its U46619 complex. The difference in position of the C-terminus for each protein is indicated in the appropriate color. (2.52 MB TIF) [file pbio.1000547.s004.tif]

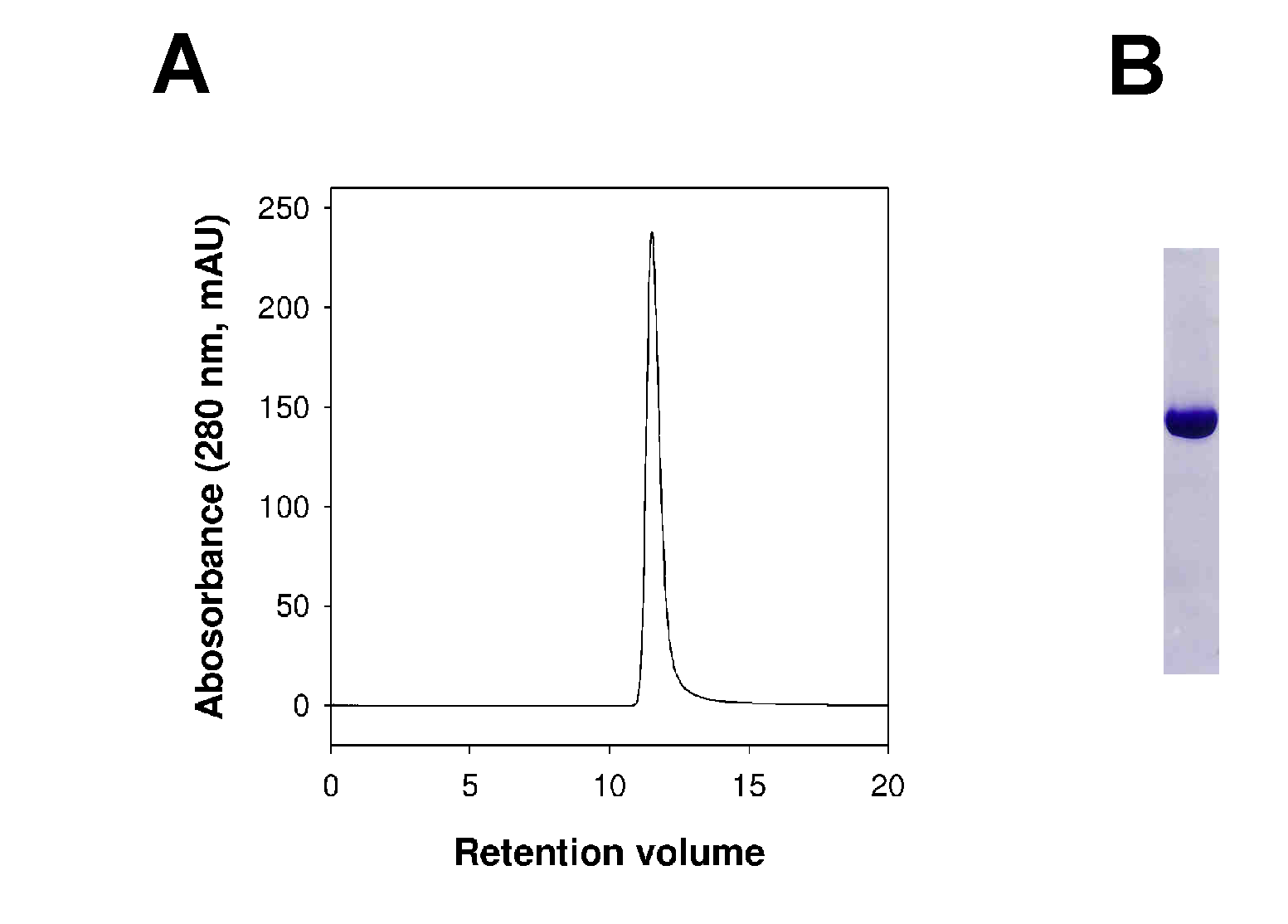

Supplement: Figure S5 — Purification of recombinant AnSt-D7L1. (A) Gel filtration chromatography of purified AnSt-D7L1 on Superdex 75. Elution buffer: 20 mM Tris HCl pH 8.0, 0.15 M NaCl. (B) SDS-PAGE gel of purified recombinant AnSt-D7L1 stained with Coomassie blue. (5.20 MB TIF) [file pbio.1000547.s005.tif]
